# Hydrodynamics of Intravitreal Injections into Liquid Vitreous Substitutes

**DOI:** 10.3390/pharmaceutics11080371

**Published:** 2019-08-01

**Authors:** Christin Henein, Sahar Awwad, Nkiruka Ibeanu, Stavros Vlatakis, Steve Brocchini, Peng Tee Khaw, Yann Bouremel

**Affiliations:** 1 Biomedical Research Centre at Moorfields Eye Hospital, NHS Foundation Trust and UCL Institute of Ophthalmology, National Institute for Health Research (NIHR), London EC1V 9EL, UK; 2 School of Pharmacy, University College London, London WC1N 1AX, UK; 3 Department of Mechanical Engineering, University College London, London WC1E 7JE, UK

**Keywords:** hydrodynamics, hyaluronic acid, vitreous, intravitreal injection, distribution, viscosity, surface tension, density

## Abstract

Intravitreal injections have become the cornerstone of retinal care and one of the most commonly performed procedures across all medical specialties. The impact of hydrodynamic forces of intravitreal solutions when injected into vitreous or vitreous substitutes has not been well described. While computational models do exist, they tend to underestimate the starting surface area of an injected bolus of a drug. Here, we report the dispersion profile of a dye bolus (50 µL) injected into different vitreous substitutes of varying viscosities, surface tensions, and volumetric densities. A novel 3D printed in vitro model of the vitreous cavity of the eye was designed to visualize the dispersion profile of solutions when injected into the following vitreous substitutes—balanced salt solution (BSS), sodium hyaluronate (HA), and silicone oils (SO)—using a 30G needle with a Reynolds number (*Re*) for injection ranging from approximately 189 to 677. Larger bolus surface areas were associated with faster injection speeds, lower viscosity of vitreous substitutes, and smaller difference in interfacial surface tensions. Boluses exhibited buoyancy when injected into standard S1000. The hydrodynamic properties of liquid vitreous substitutes influence the initial injected bolus dispersion profile and should be taken into account when simulating drug dispersion following intravitreal injection at a preclinical stage of development, to better inform formulations and performance.

## 1. Introduction

Intravitreal therapy (IVT) is one of the most frequently performed procedures across all medical and surgical specialties [1,2]. Since the advent of the first intravitreal anti-vascular endothelial growth factor (VEGF) medicine to treat age-related macular degeneration (AMD), the use of intravitreal injections has increased dramatically. The number of ophthalmic conditions being treated using intravitreal injections has also increased (e.g., diabetic macular edema (DME), retinal vascular conditions and myopic choroidal neovascularization). To treat AMD, intravitreal injections are administered monthly (e.g., ranibizumab [3]), bimonthly (e.g., aflibercept [4]), or as needed, depending on the anti-VEGF agent, pathology, and progression of the condition. These injections come at a considerable financial burden to the National Health Service (NHS) due to their frequency and cost. New anti-VEGF agents (e.g., brolucizumab [5,6]) requiring less frequent administration are emerging from development into clinical trials. Ranibizumab and aflibercept are priced between £550 and £880 per injection respectively [7]. This cost difference suggests there is significant overspending on registered drugs for AMD and that there is still an urgent need for longer-acting effective treatments with infrequent dosing at a reasonable price.

In older eyes, liquefaction of the vitreous (synchysis) occurs due to the dissociation of collagen and hyaluronan, particularly in the central area, where there is lower collagen content [8]. Vitreous liquefaction causes some loss of viscoelastic behavior [9,10]. Over the age of 70, the vitreous increases in liquid phase and there is an age-dependent increase in hyaluronan [8]. By the age of 90, the vitreous is 50% gel phase and 50% liquid phase. Understanding the viscosity of the vitreous and vitreous substitutes is important in predicting the dispersion of IVT within the vitreous cavity [11]. Diffusion is generally understood as the principle source of drug delivery in IVT. Eye movements (saccades [12,13,14]) may produce effective fluid mixing within a liquefied vitreous, in which case, convection [15] may be important for mass transport within the vitreous cavity.

Drugs for IVT undergo preclinical and clinical development before marketing approval. While some guidelines exist, the exact details of needle depth, size, and angle, injection time (injection speed with outlet velocities) and other variables can significantly differ [16,17]. For example, the location of an intravitreal injection has a direct influence on drug distribution and elimination in the vitreous [18,19]. Peak concentrations (C_max_) can vary at different vitreous locations, depending on the injection site. The drug dose is also administered based on assumptions about the location of the injection and the shape of the injected bolus volume. The amount of injected fluid reflux from the needle can be altered by varying the methods of fluid injection into the vitreous through the pars plana [16]. Drug reflux through an injection site has been reported to decrease bioavailability due to the loss of drug from the injection site [20]. Expert panel guidelines for intravitreal injections recommend the use of a 30G needle for non-viscous drugs and a wider-bore needle (27G) for more viscous intravitreal drugs. The guidelines suggest using a needle length between 13 and 18 mm and deep placement towards the central vitreous cavity to reduce vitreous reflux and incarceration (where the vitreous is trapped within the injection site) [21].

The prediction of drug distribution within the eye to avoid tissue toxicity and to determine the precise drug levels in target tissues has been a subject of modeling studies. Computational methods have been described to model drug transport following intravitreal injection from a point source [22,23,24,25,26,27] and other models have been developed to simulate drug distribution by an intraocular or periocular implant [25,28,29]. Early models primarily considered diffusion, while more recent models consider both diffusion and convection produced by the aqueous humor flow.

There has been limited consideration during preclinical studies on what the impact on dosing would be in patients with vitrectomized eyes. Vitrectomy is a surgical procedure where the vitreous is removed. People with diabetes who have suffered complications from retinal disorders tend to undergo vitrectomy [30]. Vitrectomy is a common technique that relieves the tractional forces exerted by the degenerating vitreous on the retina before cellular modeling. It has been shown to reduce macular thickness and improve visual acuity in patients with diffuse non-tractional DME [31]. Vitreous substitutes can be gas (air or expansile gas), liquids (salt solution, perfluorocarbon liquids, semifluorinated alkanes, silicone oil), or polymers, depending on the condition [32]. Patients with prior vitrectomy receiving an intravitreal injection have reduced clinical benefits due to enhanced drug clearance times (e.g., triamcinolone acetonide [33] and bevacizumab [34]). To the best of the authors’ knowledge, there is currently no regulatory requirement for testing in eyes with different vitreous statuses, despite the implications of large variations for drug distribution and bioavailability [35,36,37]. Patients receiving intravitreal injections may have had their natural vitreous removed and replaced with a vitreous substitute such as silicone oil (SO) or balanced salt solution (BSS) [38,39,40,41].

Here, we investigate the hydrodynamic effects, such as dynamic viscosity, surface tension, and volumetric density of simulated liquid vitreous and vitreous substitutes, which may influence the distribution profile of the initial bolus administered in IVT. We also examine clinically relevant injection rates and the depth of needle placement. These experimental data will inform preclinical computational modeling of IVT mixing, IVT formulation optimization, and excipient design. This study could also help inform vitreous substitute design and formulation.

## 2. Materials, Instrumentation, and Methods

### 2.1. Materials

Sodium hyaluronate (HA, 1.0–1.8 MDa) was obtained from Lifecore Biomedical (Chaska, MN, USA). BSS was obtained from Sigma Aldrich (Gillingham, Dorset, UK). SO (S500 and S1000) was purchased from VWR International Ltd (Lutterworth, Leicestershire, UK). Coomassie^®^ Brilliant Blue G250 (Merck, Darmstadt, Germany) was used for visualization.

### 2.2. Instrumentation

The rheological properties of HA and SO were measured with a Bohlin Gemini HR Nano Rheometer rotational rheometer (Malvern Instruments Ltd., Malvern, UK), whereas the rheological properties of water were measured using an m-VROC™, viscometer/rheometer-on-a-chip (Rheosense, CA, USA). Surface tension was measured with a plate reader Delta-8 (Kibron) equipped with a multi-channel microtensiometer (Delta 8, Kibron Inc., Helsinki, Finland). A 3D printed vitreous cavity model was designed using Formlabs GmbH (Berlin, Germany) and fabricated from clear resin (V4, FLGPCL04) with a resolution of 0.05 mm.

### 2.3. Methods

#### 2.3.1. Viscosity Measurements

HA solutions of different concentrations (1.0–3.0 mg/mL) were used to simulate the viscosity and rheological behavior of human vitreous in the liquid phase. HA was weighed (1.0–3.0 mg) and dissolved in water (1.0 mL) and was allowed to stir at room temperature (RT, ~25 °C) for 10–15 min, until completely dissolved. The gel was allowed to cool at RT. A swollen gel sample was placed on the Peltier plate (diameter 20.0 mm) with a gap size of 0.4 mm and 0° angle. Viscosity was measured at a range of shear rates (0.1–200 s^−1^) at 25 °C. The viscosity of water was measured using an m-VROC™ rheosense viscometer. The measuring cell was calibrated by flushing the cell through with deionized water to avoid any particle contamination from previous measurements. Viscosity was measured at ten different shear rates (0–200 s^−1^) at 25 °C. Care was taken to ensure no air bubbles were present before each run. Samples measured with the m-VROC™ rheosense viscometer (water) were filtered through a 0.22 µm microfilter before any measurements were taken.

#### 2.3.2. Surface Tension Measurements

The microtensiometer was calibrated with Milli Q^®^ water to 72.8 mN/m prior to any surface tension measurements. A validation test (water loop test) was conducted after calibration to monitor intra- and inter-channel variations, using a plate containing Milli Q water (50 μL) in each well. All obtained validation test data were within the vendor-specified surface tension range of 72.8 ± 0.5 mN/m for Milli Q water.

#### 2.3.3. Injection in Different Vitreous Substitutes

**Figure 1:**
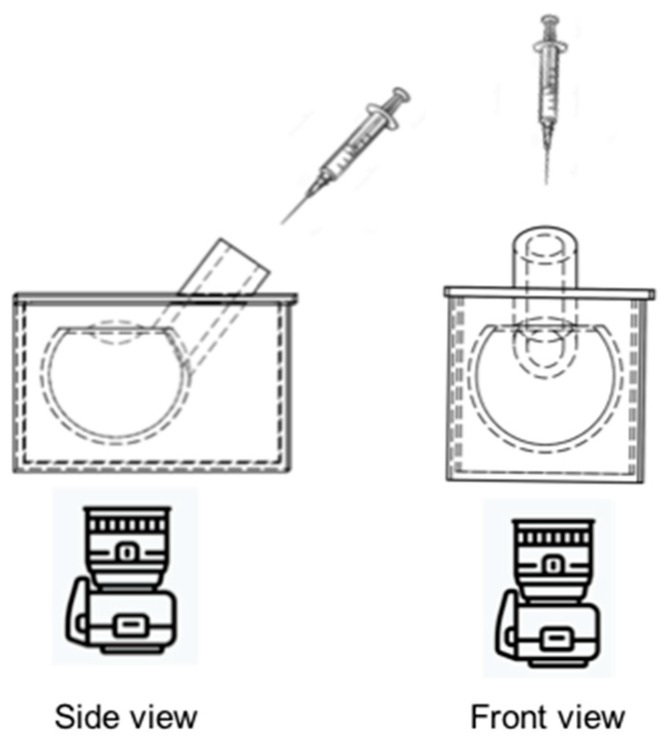
Schematic diagram of 3D printed model scaled to the geometry of an adult human eye. The vitreous cavity was completely filled with different vitreous substitutes. Dye (50 µL) was injected into the cavity via a 30G needle placed in the inlet of the model to standardize needle depth. Side (**left**) and front view (**right**) images acquired with a digital SLR camera (Canon EOS 7D). The camera was mounted with a 60 mm lens. Images were recorded at a speed of 25 Hz or every 40 ms.

A 3D printed model of the vitreous cavity was scaled to the average geometry of the adult human eye. The vitreous cavity volume was approximately 5.0 mL. The lens was 3.9 mm thick, with an anterior radius of 10.0 mm and a posterior radius of 6.0 mm. The model was fabricated from a clear resin to visualize dye dispersion within the cavity after injection. The 3D printed model (Figure 1) featured an injection inlet to accommodate a 30G needle (13 mm in length) and to standardize needle tip depth within the vitreous cavity. The needle shaft was inserted into the 3D vitreous cavity. Experiments were repeated with a shorter needle length (~6.0 mm depth) to evaluate the impact on the dye distribution when injected into different concentrations of HA. The vitreous cavity was filled with various vitreous substitutes via a 5.0 mL syringe.

To minimize diffusion as a variable from experiments, images were recorded for a maximum of 5 s. Coomassie blue dye (50 μL) with a low diffusion coefficient of 2.8 × 10^−5^ cm^2^ s^−1^ in water at 25 °C [42] was uniformly mixed in water before injection at slow and fast injection rates via a beveled stainless-steel needle (30G) attached to a 1.0 mL syringe. During the time of recording (maximum of 5 s), the Coomassie blue dye did not have time to start mixing with any vitreous substitutes, except in the case of the BSS vitreous substitute.

The Reynolds number (*Re*) is a dimensionless parameter used to characterize the ratio of inertial forces to viscous forces. Inertial forces are associated with convection and a characteristic time is *L*/*U,* where *L* is a characteristic length and *U* is the flow velocity. Viscous forces are defined by viscous diffusion and a characteristic time *L*^2^/*v*, where *v* is the kinematic viscosity (the ratio of dynamic (absolute) viscosity to density). The ratio of the characteristic times for viscous diffusion over convection leads to the definition of *Re* in Equation (1):

(1) $$Re=\frac{UL}{\nu }.$$

In the case of this experiment injecting the dye bolus with a 30G needle, *L* is the internal pipe diameter (*D* = 0.159 mm), *ν* is the kinematic viscosity of water (1 × 10^−6^ m^2^/s), and *U* is the mean flow velocity inside the needle.

For low *Re*, the viscous forces are dominant and the flow results from equilibrium between the viscous friction forces and the pressure gradient. This flow generally occurs at very low speeds or very small systems and is called laminar. At high *Re*, inertial forces dominate flow and the momentum transport is mainly driven by convection. This flow is typically turbulent. When progressing from laminar to turbulent, the flow generally progresses through a laminar–turbulent transition.

Fast injections times (below 1 s) displayed *Re* values of 189 ± 91 for all cases, whereas slow injections times (above 1 s) displayed *Re* values of 677 ± 175. Images were recorded using a digital SLR camera (Canon EOS 7D) mounted with a 60 mm lens at 25 Hz, or every 40 ms. Front and side views of the injections were recorded and processed using MATLAB software (R2017b, The MathWorks, Inc., Natick, MA, USA).

#### 2.3.4. Image Processing

**Figure 2:**
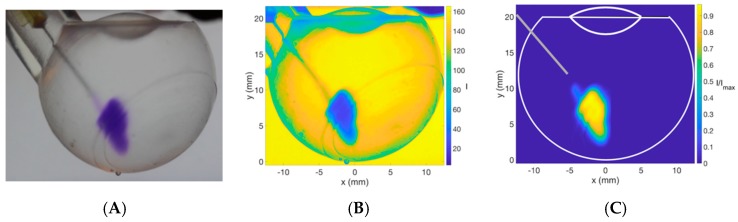
Steps taken in digital image processing. Side view photograph of injected Coomassie blue solution into sodium hyaluronate (HA, 3.0 mg/mL) with a *Re* value of 145. Raw image (**A**) converted into red, green, and blue colors (**B**) after background subtraction (**C**) using MATLAB software.

**Figure 3:**
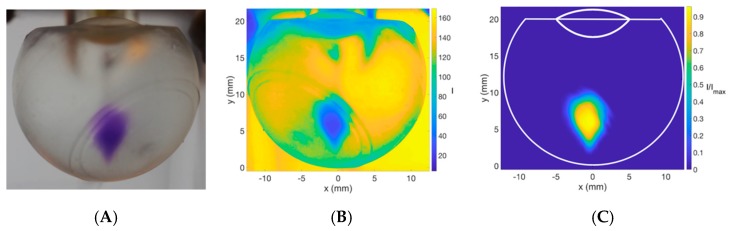
Steps taken in digital image processing. Front view photograph of injected Coomassie blue solution into sodium hyaluronate (HA, 1.0 mg/mL) with a *Re* value of 156. Raw Image (**A**) converted into red, green, and blue colors (**B**) after background subtraction (**C**) using MATLAB software.

Image acquisition and processing steps are shown in Figure 2 and Figure 3 for the side and front views, respectively. The dye area injected into the vitreous cavity is visualized and quantified at each time from image processing. This makes it possible to quantify the dispersion dynamics of injected dye into different vitreous substitutes in terms of average bolus velocity and bolus surface area. The recording started with the injection and stopped shortly after the end of injection, when the dye bolus was motionless. Image processing was conducted using MATLAB (R2017b, The MathWorks, Inc., Natick, MA, USA). Each image was first converted into red, blue and green colors using MATLAB; and the axis origin located at the bottom central part of the 3D print. Secondly, the image immediately before injection was removed from subsequent images to visualize only the dye tracer. A higher Coomassie blue dye concentration is correlated with higher dye intensity. The dye intensity of each image was normalized by the maximum dye intensity. In the present work, we were not studying the mixing of Coomassie blue dye within each vitreous substitute and we were only interested in the initial bolus of dye immediately after injection. Therefore, we did not characterize the solubility of the Coomassie blue dye in the vitreous substitute.

## 3. Results

### 3.1. Dynamic Viscosity, Surface Tension, and Density of Vitreous Substitutes

**Figure 4:**
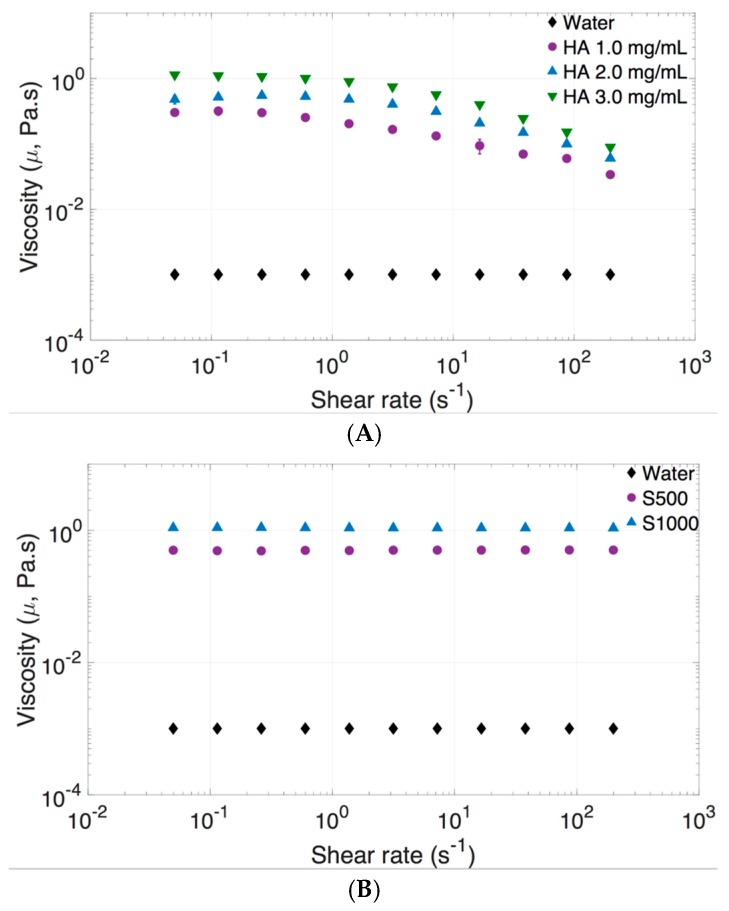
Viscosity of vitreous substitutes with an increasing shear rate (0–200 s^−1^) at 25 °C. (**A**) HA (1.0–3.0 mg/mL) exhibited a non-Newtonian and viscoelastic behavior and (**B**) SO (S500–S1000) exhibited a Newtonian fluid profile. The dynamic viscosity of water is reported on both graphs for comparison.

Physical properties of vitreous substitutes, such as viscosity, interfacial surface tension, density, and buoyancy, influence the dispersion profile of the injected bolus. The dynamic viscosity of the vitreous changes with age. In older eyes, liquefaction of the vitreous occurs, particularly in the central area, where there is a lower collagen content. Vitreous liquefaction causes loss of viscoelastic behavior. Understanding the viscosity of the vitreous and its substitutes is important in predicting the dispersion and diffusion of IVT. The dynamic viscosity values of vitreous substitutes are shown in Figure 4 and Table 1. The viscosity of HA increases with concentration, and exhibited a non-Newtonian and viscoelastic behavior. Shear-thinning behavior is demonstrated in Figure 4A, with viscosity decreasing up to a factor of over five times as the shear rate was increased. The zero-shear viscosity or the viscosity “at-rest” condition of each vitreous was used when analyzing the impact of viscosity on the dynamic dispersion profile of the solution injected, since the 3D printed model was fixed and still during experiments. SO (S500 and S1000) exhibited a Newtonian fluid profile (Figure 4B) with no change in dynamic viscosity with shear rate. Human liquid vitreous viscosity ranges from 0.1 to 0.85 Pa·s [43].

**Figure 5:**
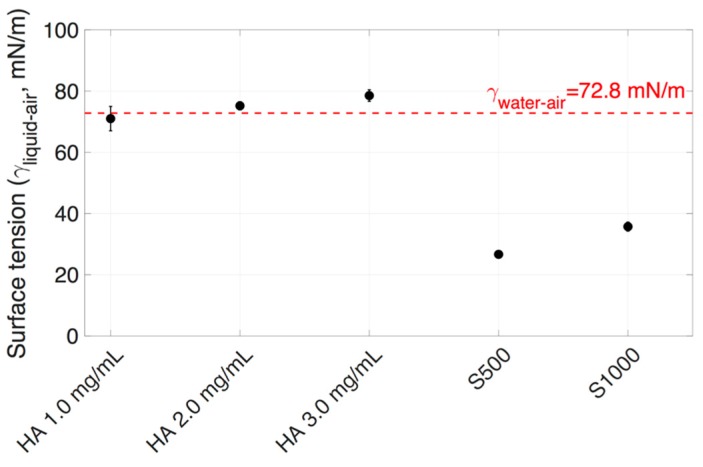
Interfacial surface tension of HA, SO (S500 and S1000), and (red dotted line) water with air at 25 °C.

Surface tension is the tendency of liquid surfaces to reduce into the minimum surface area possible. Surface or interfacial tension is defined as a force per unit of length. Some anti-VEGF IVTs contain polysorbate 20 (e.g., Avastin^®^), a surfactant that reduces the surface tension, thereby lowering the risk of protein aggregation. The surface tension of vitreous substitutes could impact the performance of IVT. The surface tension of the vitreous substitutes was characterized to understand its impact on the dispersion area of the injected dye bolus. The surface tensions of both HA and SO are shown in Figure 5 and Table 1. HA samples displayed a higher surface tension than SO samples. S500 showed a lower liquid/air surface tension of 26.7 ± 0.7 mN/m as compared to S1000, which showed a surface tension of 35.7 ± 1.5 mN/m.

**Figure 6:**
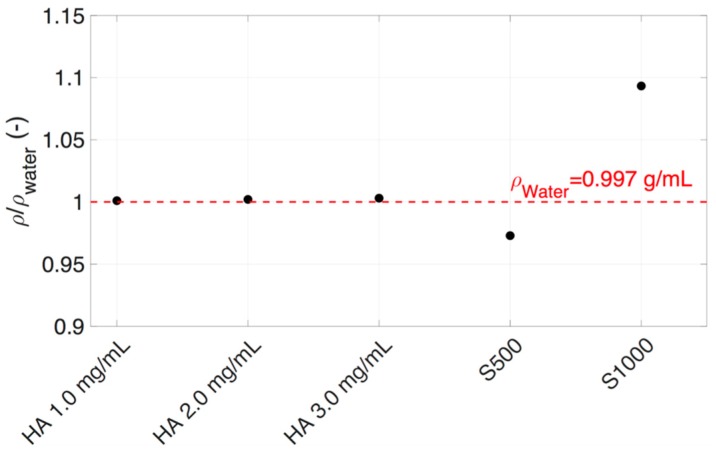
Density ratio of HA (1.0–3.0 mg/mL), SO (S500 and S1000), and (red dotted line) water at 25 °C. S1000 had the highest density and S500 the lowest density from all the vitreous substitutes tested.

The fluid density of vitreous substitutes was measured to understand the impact it had on bolus buoyancy. The volumetric density ratio of HA with water (ρ/ρ_water_) did not vary greatly between concentrations of 1.0–3.0 mg/mL, as shown in Figure 6. S500 had a lower volumetric density (0.97 g/mL) compared to water, whereas S1000 had a greater density (1.09 g/mL) (Figure 6).

**Figure 7:**
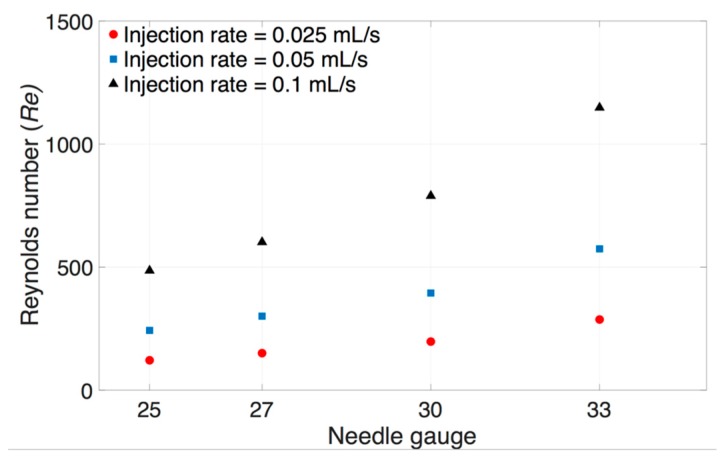
Theoretical changes in *Re* at different injection rates with varying needle gauges (25–33G). *Re* increased with smaller needle sizes for a fixed flow rate. The *Re* suggests that the flow pattern of IVT in clinically-relevant needle sizes tends to be laminar.

A range of needle gauges are used to administer IVT. Studies have shown that patients experience less pain and there is less vitreous loss when smaller sized needles are used [44]. The *Re* was calculated for different volumetric flow rates through different gauge needles (25–33G, Figure 7). *Re* defined in Equation (1) allowed us to predict the flow patterns of fluid flow within the needle. Indeed, fluid flow in a tube (or needle) is dominated by laminar flow at lower *Re* values (<2300), transitional flow (2300 < *Re* < 4000), and generally considered turbulent flow at higher *Re* values (>4000). After we defined and characterized the different physical properties of the vitreous substitutes (viscosity, interfacial surface tension, and density), as well as the injection rate in terms of *Re*, we proceeded to study the effects of each of these parameters on the dispersion dynamics of the injected liquid immediately after injection.

### 3.2. Dispersion Dynamics of Injectable Dye in Different Vitreous Substitutes

**Figure 8:**
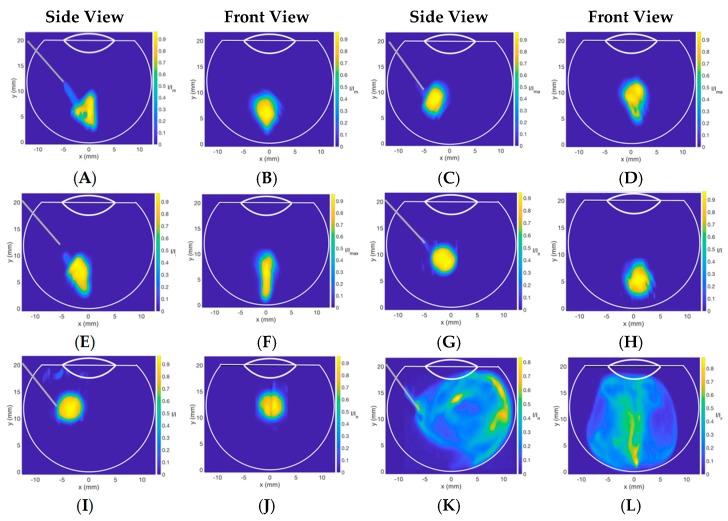
Contour plot of the dye concentration injected in different vitreous substitutes at low *Re* (189 ± 91), slow injection times (>1 s). (**A**,**B**) 1.0 mg/mL HA, (**C**,**D**) 2.0 mg/mL HA, (**E**,**F**) 3.0 mg/mL HA, (**G**,**H**) S500, (**I**,**J**) S1000, and (**K**,**L**) BSS. At slow injection rates, the injected bolus remained localized in all vitreous substitutes, thereby minimizing the stretching and spread of IVT.

**Figure 9:**
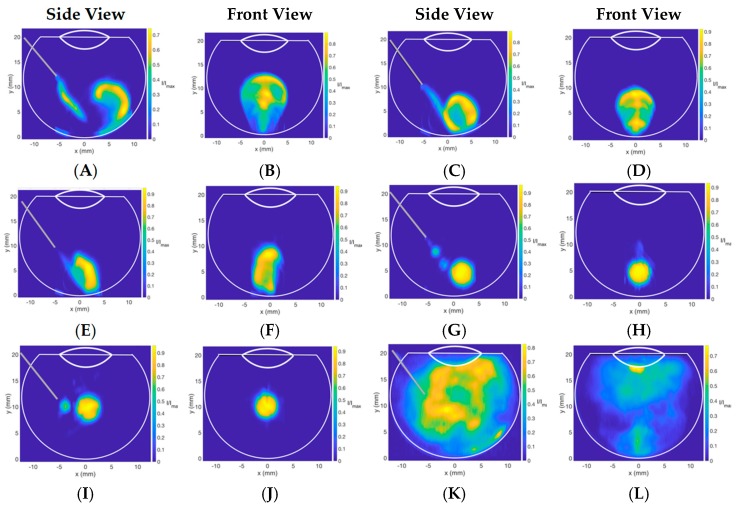
Contour plot of the dye concentration injected in different vitreous substitutes at high *Re* (677 ± 175), fast injection times (<1 s). (**A**,**B**) 1.0 mg/mL HA, (**C**,**D**) 2.0 mg/mL HA, (**E**,**F**) 3.0 mg/mL HA, (**G**,**H**) S500, (**I**,**J**) S1000 and (**K**,**L**) BSS. The fast injection rate resulted in fluid engulfment, increasing the surface area and promoting mixing and diffusion.

Image analysis performed after the injection of Coomassie blue dye (50 μL) into vitreous substitutes in the 3D printed vitreal cavity model is summarized in Figure 8 and Figure 9. At slow injection rates (*Re*: 189 ± 91), the dispersion of the Coomassie blue dye in HA (1.0–3.0 mg/mL) and SO (S500 and S1000) showed little dispersion and was locally suspended within the vitreous substitute immediately after injection (Figure 8). Injections into BSS, however, showed a wider spread with the dye impacting the posterior and lateral walls of the model, creating a recirculation motion. Injected Coomassie blue dye into HA had an ellipsoidal distribution compared to dispersion in SO, which had a more spherical shape at slow injection rates. This can be explained by the large range of surface tensions of the different vitreous substitutes shown in Figure 3 and Table 1. Differences in air/fluid surface tension between water and the vitreous substitutes ranged from 3 to 8% for HA solutions (1.0–3.0 mg/mL), while increasing to 51% and 63% for S1000 and S500 respectively. The larger the difference in interfacial surface tension, the more spherical is the injected bolus. The spread of the injected bolus was different when water was injected into BSS; the dye distribution was non-uniform. The stretched lamellae of dye denote the quasi-absence of interfacial surface tension between the dye injected and the vitreous substitute.

At the faster injection rate in Figure 9, we observed engulfment of the injected dye in different vitreous substitutes. Engulfment is part of the micro-mixing model called the engulfment–deformation–diffusion model, where deformations of structures are embedded in a turbulent fluid [45]. The term engulfment may also be used in the current set-up, where a liquid is injected into more viscous fluids and is embedded in a recirculation motion. At faster injections rates (*Re*: 677 ± 175), the injected Coomassie blue dye impacted the posterior part of the model, engulfing the dye along the walls of the model. Injection into lower concentrations of HA (1.0 mg/mL) (Figure 9A and B) demonstrated larger engulfment compared to higher concentrations (3.0 mg/mL) (Figure 9E and F). The lower the viscosity of the HA solution, the larger the engulfment of injected dye. An increase in engulfment (260%) was seen with 3.0 mg/mL (1.14 Pa·s) from 1.0 mg/mL (0.30 Pa·s). The dispersion profiles of injections into SO at high *Re* values were similar to profiles seen in low *Re* values, except for the occurrence of satellite droplets due to the faster injection rate. Fast injection into BSS resulted in a quicker and longer recirculation motion within the vitreous cavity compared to a slower injection rate due to the additional kinetic energy delivered by the ejection of dye from the needle. Faster injection rates increased mixing by promoting dye convection, which was denoted by the larger area of normalized dye with high intensity. A video of injection into 2.0 mg/mL HA at high *Re* is provided as a Supplementary File online (Video S1).

**Figure 10:**
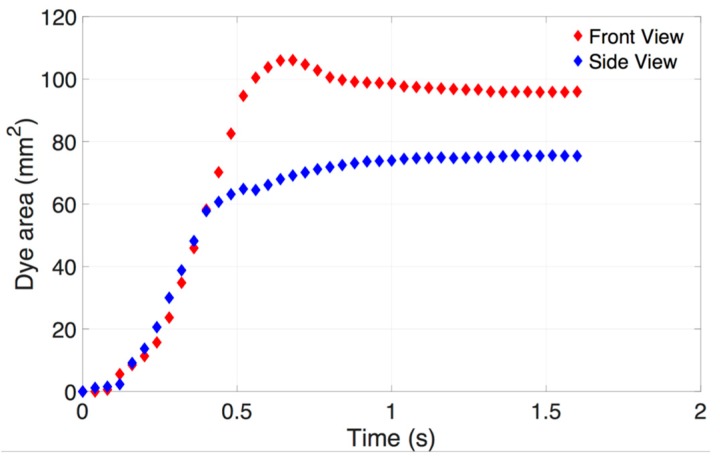
Evolution of dye area against time in the case of fast injections (*Re* = 589) into the HA vitreous (1.0 mg/mL). The dye area was measured from images taken from a digital SLR camera at 25 Hz, or every 40 ms. The dye area reached a maximum area of 106 mm^2^ from the front view and 72 mm^2^ from the side view. This indicates the bolus is not perfectly spherical in shape and this assumption should be avoided when modeling IVT in liquid-phase vitreous.

The evolution of dye area in 1.0 mg/mL HA vitreous substitutes against time, reached a maximum area of 106 mm^2^ and 72 mm^2^ from the front and side view respectively (Figure 10). This indicates the bolus is not spherical in shape and this assumption should be avoided when modeling IVT in liquid-phase vitreous. Only dye corresponding to a grey scale intensity above 10 (maximum grey scale intensity of 256) was used to calculate the dye area, to remove any noise that may be present after subtraction of the background image. The dye area increased monotonically to the end of injection ~0.68 s (front view) and ~0.92 s (side view). In this particular case, the dye was seen to spread more perpendicularly to the direction of injection than parallel to it when injected into HA (1.0 mg/mL). The values of dye area reaching the plateau from the side and front view were reported on each consecutive graph. We divided the injection time into fast (*Re*: 677 ± 175) and slow (*Re*: 189 ± 91) injection rates to reproduce clinically relevant injection rates. These *Re* values correspond to the flow inside the needle. Additional *Re* can be calculated for the dye movement within the vitreous substitute (*Re*_bolus_) to quantify the effects of viscous and inertial forces. We considered the averaged velocity of displaced dye and the average length of the bolus. Averaged bolus velocity was calculated from the averaged bolus length (i.e., square root of the dye area) in time.

**Figure 11:**
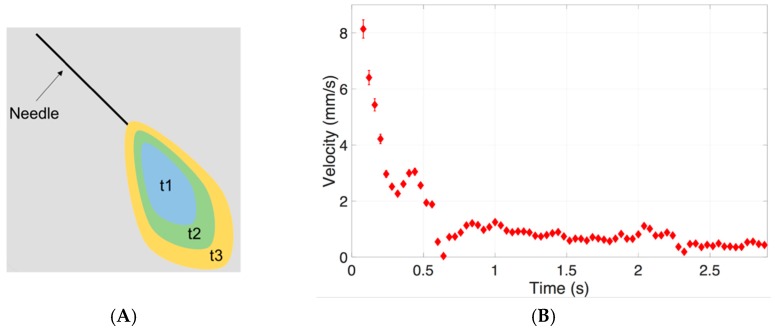
(**A**) Schematic representation of dye area dispersion in vitreous substitute at different time points and (**B**) a typical velocity profile of dye injected into S1000 at a slow injection rate (*Re:* 189 ± 91). The velocity of the injected bolus was calculated from changes in bolus length (i.e., square root of the dye area) in time.

The *Re* of the bolus movement (*Re*_bolus_) through the vitreous substitutes was calculated from the dye area at serial time points. The velocity of the injected bolus was calculated from changes in the bolus length between two different time points. A schematic representation of the dye area dispersion in time is shown in Figure 11A. The velocity of the injected bolus in S1000 is shown in Figure 11B. At the start of injection, the velocity reached a value of 8.0 mm/s, before slowing down to approximately 0.5 mm/s.

**Figure 12:**
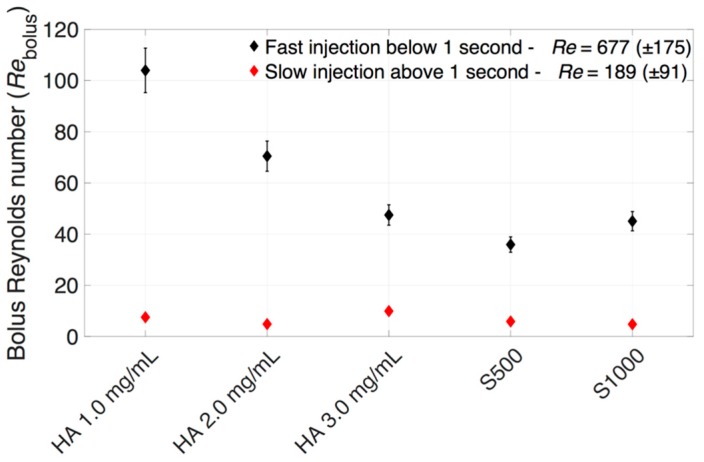
Average *Re*_bolus_ within different vitreous substitutes at (black diamonds) fast and (red diamonds) slow injection rates.

The averaged *Re*_bolus_ for dye injected into the different HA and SO vitreous is shown in Figure 12. For slow injection rates (*Re*_bolus_ = 4.8–10), the viscous forces dominated the transport of the dye, while at fast injection speeds, a maximum *Re*_bolus_ of 104 was observed, indicating that viscous forces still dominated, albeit convection may also be playing a role, as shown by the engulfment in Figure 9.

**Figure 13:**
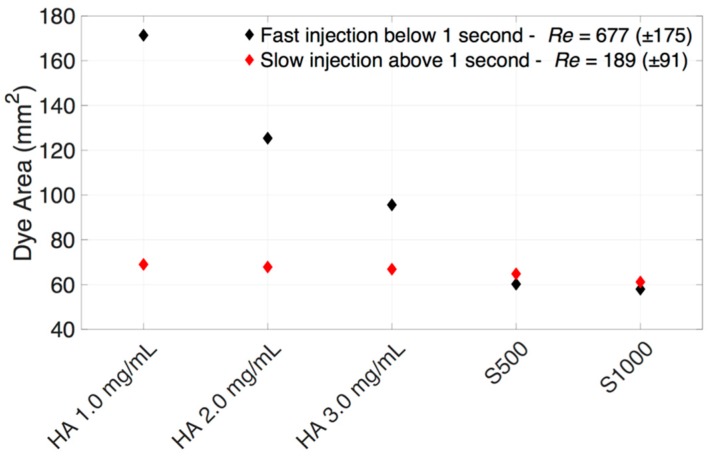
Impact of (black diamonds) high and (red diamonds) low *Re* on the distribution of dye injected into HA (1.0–3.0 mg/mL) and SO (S500 and S1000). The surface area of the dye was considerably reduced with increasing HA concentrations at fast injection rates compared to slow injection rates. The bolus was more localized in all vitreous substitutes at slow injection speeds.

The summation of front and side view dye areas in different vitreous substitutes is shown in Figure 13. A larger dye area was correlated to larger stretching of the dye bolus. Larger areas for a given volume promote mixing by increasing the interfacial tension available for diffusion. For HA samples (1.0–3.0 mg/mL), fast injection rates consistently increased the dye area compared to low injection rates, indicating the effect of extra momentum from the fast injection rate on dye stretching. For example, the dye area in 1.0 mg/mL HA increased by 148% from 69 mm^2^ at a slow injection rate to 171 mm^2^ at a fast injection rate. There was minimal difference between the dye areas in SO at fast and slow injection rates. At fast injection rates, satellite droplets were observed in SO, signifying the effects of interfacial surface tension in keeping the dye bolus spherical, countering the effects of dye stretching. These satellite droplets were not as well defined as large droplets, resulting in a slightly lower dye area in SO at a fast injection rate and a limitation of the processing technique.

**Figure 14:**
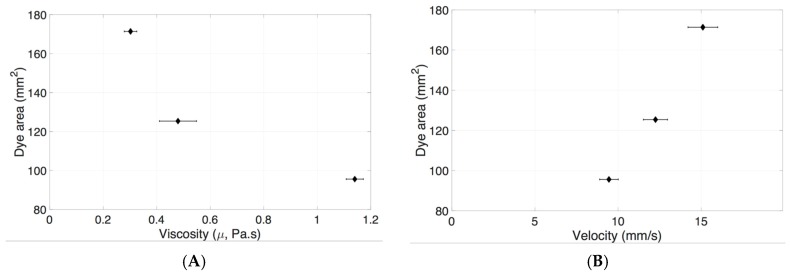
Correlation between bolus dye areas in HA vitreous substitutes. (**A**) Shear-zero viscosity and (**B**) velocity when dye was injected at high *Re* (677 ± 175).

The dye surface area was considerably reduced with increasing HA concentrations at fast injection rates compared to slow injection rates. The interfacial surface tensions of HA (1.0–3.0 mg/mL) were similar, with a maximum difference of 10% (Figure 5 and Table 1). A maximum difference of 260% was seen with varying viscosities of HA (0.30 ± 0.02 Pa·s to 1.14 ± 0.03 Pa·s, Figure 4 and Table 1). The increase in HA viscosity correlated with a decrease in surface area (Figure 14A). This is due to the effect of viscosity dissipating kinetic energy into heat, slowing down the dye velocity in the vitreous substitute, hence reducing the size of engulfment. A higher dye velocity was seen with a larger dye area in HA (Figure 14B). The viscosity of the vitreous substitute plays a major role in determining the total surface area of the bolus injected and this property should be considered carefully when developing vitreous substitutes.

**Figure 15:**
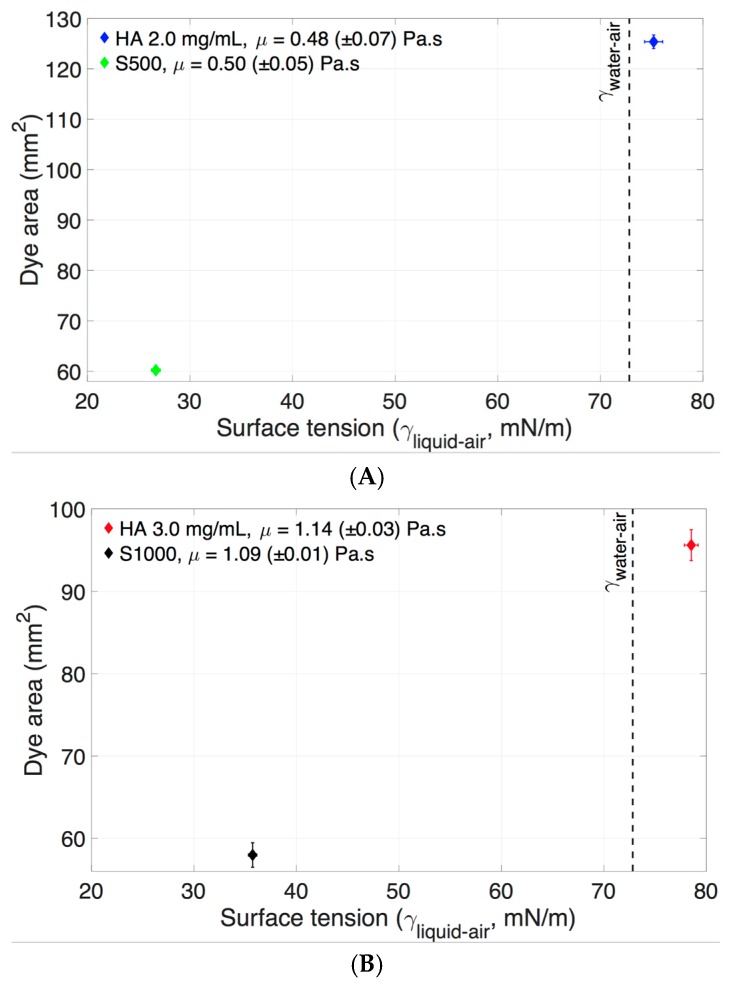
The effect of surface tension on dye area at high *Re* into vitreous substitutes of equivalent viscosities. The higher surface tension difference between air/vitreous substitutes and air/water resulted in a lower injected dye dispersion area. (**A**) HA (2.0 mg/mL) and S500, (**B**) HA (3.0 mg/mL) and S1000 and (vertical black dotted line) the surface tension of water and air.

The other important parameter to consider is the effect of the interfacial tension of vitreous substitutes on dye area. Dye area was greater when there was a smaller difference between the vitreous substitute and bolus solution air/fluid surface tension (Figure 5 and Table 1). HA (2.0 mg/mL) and S500 displayed similar viscosities but different interfacial (liquid/air) surface tensions (75.2 ± 1.3 mN/m or 3% difference from water and 26.7 ± 0.7 mN/m or 63% difference from water respectively). At fast injection rates, the dye surface area was 125.4 mm^2^ in HA (2.0 mg/mL), as compared to 60.2 mm^2^ in S500 (Figure 15A, a reduction of ~52%). Likewise, HA (3.0 mg/mL) and S1000 had similar viscosities but different interfacial (liquid/air) surface tensions (78.5 ± 1.9 mN/m or 8% difference from water and 35.7 ± 1.5 mN/m or 51% difference from water, respectively). The dye area was 95.6 mm^2^ in HA (3.0 mg/mL), as compared to 58 mm^2^ in S1000 (Figure 15B, a reduction of ~39%). The effect of surface tension prevented the injected liquid from stretching, and the extra fluid momentum broke down the stream of injected liquid, producing satellite droplets, therefore further reducing the surface area of the injected liquid. The surface areas of the injected liquid at high and slow injection rates (Figure 13) were similar in the SO vitreous, due to the fact that the effects of surface tension prevented the liquid engulfment and instead broke into satellite droplets.

**Figure 16:**
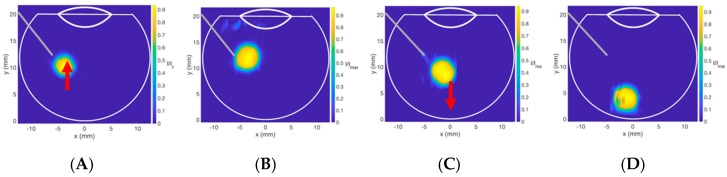
Contour plots of Coomassie blue dye injected into S500 and S1000. (**A**,**B**) Upward movement showing the buoyancy of injected bolus into S1000 (density: 1.09 g/mL) and (**C**,**D**) downward movement of the injected solution into S500 (density: 0.97 g/mL).

The last parameter to consider is the effect of density on bolus buoyancy. A fluid with a lower volumetric density than the vitreous substitute into which it is injected will tend to rise. Inversely, injected fluids with higher volumetric density will sink to the bottom of the vitreous cavity. Consideration should be given to the density of IVT in relation to the vitreous and vitreous substitutes in which they are injected. The bolus injected into S1000 exhibited buoyancy, as the volumetric density of S1000 was 9% heavier than the injected dye. S500 had a volumetric density 3% lighter than the bolus solution, so the bolus settled on the posterior wall (Figure 16).

**Figure 17:**
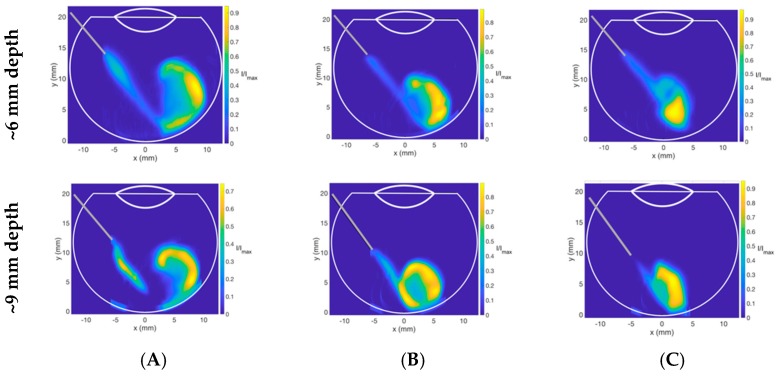
Side view contour plots of Coomassie blue dye injected into (**A**) 1.0, (**B**) 2.0 and (**C**) 3.0 mg/mL HA at high *Re* (677 ± 175) at needle depths of (upper panel) approximately 6 mm and (lower panel) 9 mm. Deeper needle placement may be preferable for delivering IVT.

Expert panel guidelines recommend a needle length of 13 to 18 mm needle into the vitreous cavity, suggesting that different needle lengths may be used for IVT, and also recent studies show variation in the depth of needle placement between IVT administration amongst healthcare practitioners [46]. The study of the needle depth inside the vitreous cavity on drug dispersion is therefore crucial. For the present study, a 30G needle (13 mm in length) was used. However, a 2 mm needle length was located within the yellow hub, leaving 11 mm for placement into the vitreous cavity. The dispersion dynamics of Coomassie blue dye for two different needle depths of approximately 6 and 9 mm inside the vitreous cavity are shown in Figure 17. Injections at shallower needle depths (Figure 17 upper panel) demonstrated larger dye areas for all HA concentrations compared to deeper needle placement (Figure 17 lower panel), with an average of 36% increase in area. Due to the length of the needle within the vitreous cavity being 35% shorter on average when the needle is not fully inserted, the dye reached the posterior aspect of the cavity with a lower velocity, reducing the amount of engulfment for all HA concentrations. For 3.0 mg/mL HA, the dye did not reach the back of the eye, showing that at a relatively high viscosity, short needle placement may prevent the bolus from reaching the retina, which is the target of IVT. Deeper needle placement may therefore be preferable for delivering IVT, to ensure that the drug reaches the retina, especially if the vitreous has a slightly larger viscosity.

## 4. Discussion

Intravitreal drugs are injected into the vitreous cavity as suspensions or solutions via the pars plana using 27 or 30G needles. Advances in bio-nanotechnology means there are emerging controlled release systems for drug delivery to the posterior segment in an injectable form. Understanding the convection of the initial bolus, as well as diffusion and buoyancy, are important for predicting drug biodistribution at the preclinical stages of drug development.

In summary, simulated intravitreal injections using a 30G beveled needle and a 3D model of the vitreous cavity filled with vitreous substitutes showed that the viscoelastic properties of HA reduced the area of distribution of the injected solution at higher concentrations at fast injection rates. The volumetric density of a vitreous substitute in relation to the injected solution determined the buoyancy of the bolus. Denser than water vitreous substitutes, such as standard SO (S1000), resulted in the buoyancy of injected bolus rising up to the meniscus. The use of vitreous substitutes with densities lower than water, such as S500, settled at the bottom of the posterior pole of the vitreous cavity. We showed that when there is little difference in the air/fluid surface tension between the vitreous substitute and the injected liquid (in this study, <8%), a fast injection rate results in fluid engulfment, increasing the surface area and promoting both mixing and diffusion. The distribution of the injected bolus is driven by viscous forces and the initial fluid momentum stretching the bolus during the injection. At fast injection rates, a large difference in surface tension (in this study, >51%) between the air/vitreous substitute and air/water prevented the fluid from engulfing. This resulted in the shedding of droplets of different sizes, leading to smaller surface areas and therefore reducing the surface available for mixing and diffusion. Many of these parameters are affected by temperature. We conducted this study at RT, whereas the temperature is approximately 34 °C and 25 °C in vivo before and during vitrectomy, respectively [47]. However, the *Re* value remained low when corrected for posterior segment physiological temperatures, suggesting laminar flow behavior.

Injection into BSS resulted in the largest dye distribution area and fastest mixing, as expected. When the dye was injected into BSS, it impacted the cavity wall and heterogeneously formed spreading lamella in all directions. Our method using a vitreous cavity model demonstrates the importance of using relevant ocular globe and lens geometry on the dispersion of the injected solution inside the cavity. The bolus was localized at the posterior pole of the eye for all vitreous substitutes (except for BSS) at the end of the injection. The bolus did not reach the posterior wall of the model at slow injection rates and at fast injection rates for all SO and in the case of 3.0 mg/mL HA when the needle depth inside the vitreous cavity was reduced by 35%. The bolus impacted the vitreous cavity wall, creating larger engulfment in HA samples with lower concentrations at fast injection rates. The bolus exhibited buoyancy when the vitreous substitute was denser than the bolus solution or settled to the bottom of the cavity when the vitreous substitute was lighter.

The simulation of intravitreal injections is complex. Although physiologic-based pharmacokinetic models are limited due to simplification and empiricism, they are still very important in the preclinical development of new IVT. Simple volumetric scaling of experimental results from one species to another is not always appropriate. Compartmental modeling using computational fluid dynamics using anatomically relevant models allows biodistribution predictions to be made based on boundary conditions, mass, flow pressure, and concentration applied to various tissue surfaces. However, many of these models may not consider initial injection rate, lens status, wall impact, and assume a spherical-shaped bolus of a specified starting surface area. In this study, we showed how the injection rate, viscosity, and surface tension influence the surface area of the injected bolus. In addition to these parameters, buoyancy affects the time evolution of the injected bolus. During the time of recording (maximum of 5 s), the Coomassie blue dye did not have time to start mixing with any vitreous substitutes, except in the case of BSS. Therefore, diffusion in our experiments was negligible during this time frame and the surface area reported reflects the combination of viscosity, convection, and surface tension of the initial injected bolus. The diffusivity of the bolus is also influenced by drug properties, such as particle size, polydispersity, and surface charge. Larger drug molecules have slower diffusivity due to entanglement or interaction with the biopolymeric component of the gel vitreous [13]. Increasing the hydrodynamic radius of therapeutic proteins has been suggested as a method to extend vitreal half-life, to enable less frequent dosing [48].

To the best of our knowledge, the distribution of the initial bolus of intravitreal solutions at clinically relevant speeds and volumes of injection into vitreous substitutes has not been previously described. An important strength of our experimental model is that it reflects the ocular globe, including the presence of a lens, which influenced fluid dynamics, particularly when dye was injected into BSS. The dye impacted the posterior surface of the lens and re-circulated into BSS. The lens status of patients receiving intravitreal injections may influence drug flow dynamics in an ageing liquefied vitreous or in post-vitrectomized eyes with BSS as a vitreous substitute.

Following intravitreal injections, the intraocular pressure (IOP) is transiently raised because globe tissues exhibit elastic properties. Our model was fabricated from a hard resin and the non-pressurized cavity was not susceptible to an increased volume, thus removing any hydraulic pressure factor in our model. After delivery of the injection, the needle remained in place for the entire duration of image acquisition. It has been previously observed that once the needle is withdrawn, a portion of the injected material may flow away from the region of the injection track along the path created by the needle [16].

A limitation of our study is that thermal convection across the posterior segment was not simulated in our model. However, this is unlikely to significantly affect the initial injected bolus surface area. Surface area calculations from 2D images work extremely well for spheres but 3D imaging would provide more accurate quantification of the bolus distribution. Despite using 2D imaging for the determination of the surface area of the bolus, it is apparent that computational models assuming a perfectly volumetric sphere bolus starting point underestimate the actual surface area of the injected bolus. Underestimating the starting point impacts the predicted surface area for drug molecule diffusion and drug clearance.

Further work should aim to characterize the impact of protein, salts, ascorbic acid, and cross-linked collagen on the hydrodynamics of intravitreal injections at physiological temperatures. This will enable preclinical modeling of drug dispersion and mixing in vitrectomized and non-vitrectomized eyes. Such modeling, in conjunction with pharmacokinetic/pharmacodynamic (PK/PD) and in-vitro in-vivo correlation (IVIVC) studies could better inform drug dosing and regimens in different vitreous and lens statuses.

## 5. Conclusions

Our study highlights key parameters and physical characteristics, which should be considered when designing or creating a vitreous substitute. Improved quantitative proteomic analyses of the vitreous through mass spectrometry-based methods have helped identify future therapeutic targets or agents to be added to vitreous substitutes. However, viscosity, volumetric density, and surface tension of the vitreous substitutes should also be considered. Properties influencing the convection of initial bolus and the diffusion of injected agents into novel vitreous substitutes should be simulated at a preclinical stage of development.

## Figures and Tables

**Table 1:** Summary of viscosity and surface tension measurements of vitreous substitutes.

Vitreous Substitute	Viscosity (Pa·s)	Surface Tension (Liquid/Air) (mN/m)
Distilled water (control)	0.0010 ± 0.0001	72.8 ± 0.1
Sodium hyaluronate (HA)		
1.0 mg/mL	0.30 ± 0.02	71.0 ± 4.0
2.0 mg/mL	0.48 ± 0.07	75.2 ± 1.3
3.0 mg/mL	1.14 ± 0.03	78.5 ± 1.9
Silicone Oil (SO)		
S500	0.50 ± 0.05	26.7 ± 0.7
S1000	1.09 ± 0.01	35.7 ± 1.5

## Supplementary Materials

The following are available online at https://www.mdpi.com/1999-4923/11/8/371/s1, Video S1: Fast injection rate into sodium hyaluronate (HA) 2.0 mg/mL at *Re* = 677 ± 175.
